# Advances in the DNA methylation hydroxylase TET1

**DOI:** 10.1186/s40364-021-00331-7

**Published:** 2021-10-16

**Authors:** Wenzheng Liu, Guanhua Wu, Fei Xiong, Yongjun Chen

**Affiliations:** Department of Biliary and Pancreatic Surgery, Tongji Hospital, Tongji Medical College, Huazhong University of Science and Technology, 1095 Jiefang Avenue, Wuhan, 430030 Hubei China

**Keywords:** Ten-eleven translocation 1, DNA demethylation, Stem cells, Immunity, Clinical disease, Malignant tumours, Leukaemia, Breast cancer, Lung cancer, Hepatocellular carcinoma, Cholangiocarcinoma, colon cancer, Pancreatic cancer, Prostate cancer, Cervical cancer, Metabolism, Neurological system

## Abstract

**Background:**

The ten-eleven translocation 1 (TET1) protein is a 5-methylcytosine hydroxylase that belongs to the TET protein family of human α-ketoglutarate oxygenases. TET1 recognizes and binds to regions of high genomic 5′-CpG-3′ dinucleotide density, such as CpG islands, initiates the DNA demethylation program, and maintains DNA methylation and demethylation balance to maintain genomic methylation homeostasis and achieve epigenetic regulation. This article reviews the recent research progress of TET1 in the mechanism of demethylation, stem cells and immunity, various malignant tumours and other clinical diseases.

**Conclusion:**

TET1 acts as a key factor mediating demethylation, the mechanism of which still remains to be investigated in detail. TET1 is also critical in maintaining the differentiation pluripotency of embryonic stem cells and plays anti- or oncogenic roles in combination with different signalling pathways in different tumours. In certain tumours, its role is still controversial. In addition, the noncatalytic activity of TET1 has gradually attracted attention and has become a new direction of research in recent years.

## Background

DNA methylation, an important epigenetic modification, has a profound impact on genome stability, transcription and development. This term specifically refers to the conversion of a methyl group onto the C5 position of 5′-CpG-3′ dinucleotides to form 5-methylcytosine (5mC), which is catalysed by DNA methyltransferase (DNMT) with S-adenosyl methionine (SAM) as the active methyl donor. DNA methylation does not change the sequence of DNA bases and has reversible biological properties, namely, the reduction of 5mC to 5-cytosine (5C) after DNA methylation is catalysed by “demethylase”. Cells rely on DNA methyltransferases and demethylases together to maintain genomic methylation homeostasis. Imbalances in genomic methylation homeostasis contribute to various diseases, including cancer.

The mechanism of DNA demethylation is considerably complicated; it has been classified as active demethylation and passive demethylation and involves various 5-methylcytosine catalases. Active DNA demethylation refers to an enzymatic process that removes or modifies the methyl group from 5mC. In contrast, passive DNA demethylation refers to loss of 5m0C during successive rounds of replication in the absence of functional DNA methylation maintenance machinery. The ten-eleven translocation (TET) protein family is a key catalytic protein that mediates DNA demethylation and plays an important role in both active and passive demethylation processes. The human TET protein family has three members, TET1, TET2 and TET3, of which TET1, a 5mC hydroxylase, is closely associated with tumorigenesis and progression through its abnormal expression and/or function.

## Basic characteristics of TET1

TET1 was the first to be defined in the human TET protein family and was identified as a fusion protein by Ono R et al. in a study of a special patient with acute leukaemia [1]. TET1 was named the ten-eleven translocation (TET) protein based on the presence of t (10; 11) (q 22; q 23) ectopically in this patient. Subsequently, Tahiliani et al. [2] discovered the TET family of proteins in mammals in 2009 and later was the first to identify that TET1 could catalyse the hydroxylation of 5mC in vitro. TET1 has a cysteine-rich domain (CD) at the C-terminus and a Cys-Xaa-Xaa-Cys (CXXC) domain at the N-terminus. Multiple β-chains form a double-stranded b-helix (DSBH) with 2OG-Fe^2+^-dependent oxidase characteristics together with a CD domain to form the catalytic domain of TET1 [3–5], allowing TET1 to recognize and bind unmodified, 5mC-modified and 5-hydroxymethylcytosine (5hmC)-modified CpG-DNA [6, 7].

## TET1 initiates the process of DNA demethylation

In the mammalian genome, 5mC is mainly distributed in CpG dinucleotides, and approximately 70–80% of CpGs are methylated [8]. TET1 is an active 5mC hydroxylase that hydroxylates 5mC to 5hmC in vivo and in vitro, thus initiating the DNA demethylation process [9]. Three main mechanisms of TET1-mediated demethylation have been described.

### TET1 protein prevents DNA from maintenance methylation

**Fig. 1:**
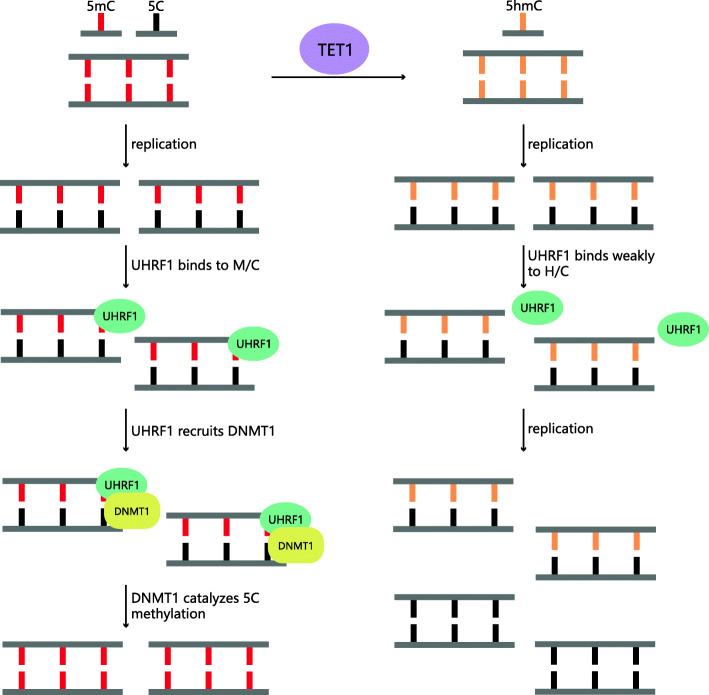
TET1 prevents DNA from maintenance methylation. TET1 catalyzes initial DNA strand methylation site hydroxylation to 5hmC, which lead to the inability of UHRF1 binding to the daughter strand and DNMT1 catalyzing 5C to 5mC. Thereby preventing the maintenance of DNA methylation and thus passively eliminating 5mC by replication

In DNA replication, hemimethylated (M/C) and hemihydroxymethylated (H/C) CpG dinucleotides are produced transiently, where only the parental strand is modified while the daughter strand remains an unmodified cytosine. DNA maintenance methylation is mediated by DNA methyltransferase 1 (DNMT1) and ubiquitin-like with PHD and ring finger domains 1 (UHRF1). UHRF1 binds to the hemimethylated CG site through the SAD/SRA structural domain and recruits DNMT1, which interacts with the CG site to maintain the DNA methylation pattern during cytokinesis [10]. TET1 catalyses initial DNA strand methylation site hydroxylation to 5hmC. In vitro experiments show that DNMT1 is 50-fold more active intrinsically against M/C substrates than against H/C substrates and that UHRF1 binds 10-fold less well to H/C substrates than to M/C substrates [11, 12]. The above studies show that in vivo, TET1 prevents UHRF1 and DNMT1 from binding to the daughter strand by catalysing the parental strand 5mC to 5hmC, thereby hindering the maintenance of DNA methylation and thus passively eliminating 5mC by replication. However, Kubosaki A [13] et al. stably transferred hydroxymethylated plasmids and methylated plasmids into human cells separately and found that both were maintained to the same extent for methylation during cell replication and division, suggesting that 5hmC does not completely hinder the maintenance of methylation in cells and that the molecular mechanism for the presence of hemihydroxymethylated CpG recognition has not been explored (Fig. 1).

### TET1-mediated active demethylation in DNA repair

In contrast to the extent of passive dilution of DNA methylation dependent on DNA replication, active demethylation is not dependent on DNA replication and is closely related to the oxidative activity of TET1 on 5mC and base excision repair (BER). TET1 first hydroxylates 5mC to 5hmC as a demethylation intermediate, and the next conversion of 5hmC may involve two aspects.

On the one hand, studies on DNA demethylation point to a mechanism involving TET and thymidine-DNA glycosylase (TDG) [7]. Current studies suggest that this pathway is formed by gradually catalysed oxidation of 5mC by TET1 to form the TDG-dependent BER substrates 5-formylcytosine (5fC) and 5-carboxylcytosine (5caC), followed by the replacement of 5mC with C to restore the unmethylated DNA sequence [6]. TDG is a protein which mediates DNA repair, that recognizes various cytosine and 5mC base derivatives in DNA [14]. TDG has long been a focus of biochemical research, as T·G or hmU·G mismatches are known TDG substrates, and TDG can excise base T and replace unmodified C during genomic T·G mismatches [15]. This biochemical pathway is supported by TET and TDG knockout mice and embryonic stem cells [16]. The involvement of the BER system implies the generation of apurinic/apyrimidinic (AP) sites [14]. The AP site is first cleaved by apurinic aprimidinic endonuclease/Redox factor-1(APE1/Ref-1), an enzyme that produces DNA single-strand breaks followed by DNA gap filling and ligation by activating poly ADP-ribose polymerase 1, X-ray repair cross complementary protein 1 (XRCC1), DNA ligase 3 (LIG3), and DNA polymerase b (POLb) [16]. Thus, TET1 and TDG initiate DNA demethylation by oxidizing and excising 5mC from DNA.

On the other hand, Guo,J et al. [17] revealed in virus-transfected human cells and mouse brains that 5hmC can be deaminated by activation-induced deaminase (AID) and apolipo protein B mRNA-editing enzyme-catalysed polypeptide-like (APOBEC) protein. APOBEC catalyses deamination of 5-hydroxymethyluracil (5hmU), which is then recognized and excised by TDG [18]. Then, the site is converted to cytosine via the BER pathway, which achieves DNA demethylation. These deamination-mediated demethylation pathways may also involve the DNA damage response protein growth arrest and DNA damage inducible 45 (GADD45), and methyl-CpG-binding domain 4 (MBD4) may even be used as a glycosylase alternative for excision of T-G mismatches [19–21]. In human cells cultured in vitro, AID/APOBEC deaminase specifically promoted 5hmC demethylation but had no significant effect on 5mC. In the adult mouse brain, AID contributed to endogenous 5hmC removal. In addition, TET1 and AID facilitate region-specific demethylation of neurons in the dentate region [10]. This mechanism has been found in stonefish [20], neurons [17], human tumour cells [22] and primordial germ cells [23]. However, the mechanism remains controversial, with some studies showing that AID does not interact with double-stranded DNA (dsDNA) and that the 5mC responsiveness to AID deamination in vitro is much lower than that of nonmethylated cytosine [24]. Christopher S. Nabel et al. [25] found that the activity of purified AID/APOBEC at 5mC was greatly reduced relative to its conventional substrate cytosine, and all AID/APOBEC family members preferentially interact with unmodified cytosine. Furthermore, after AID/APOBEC overexpression, no significant expression of the deamination product of 5hmC was detected in genomic DNA, whereas intermediates in the iterative oxidative pathway were readily found in genomic DNA and their formation was not reduced by deaminase overexpression. After a series of reactivity studies of the intermediates, it was found that their spatial volume became increasingly unfavourable for deamination, and therefore this study concluded that the spatial requirement for cytosine deamination is an intrinsic obstacle for deaminase to perform its DNA demethylation function.

**Fig. 2:**
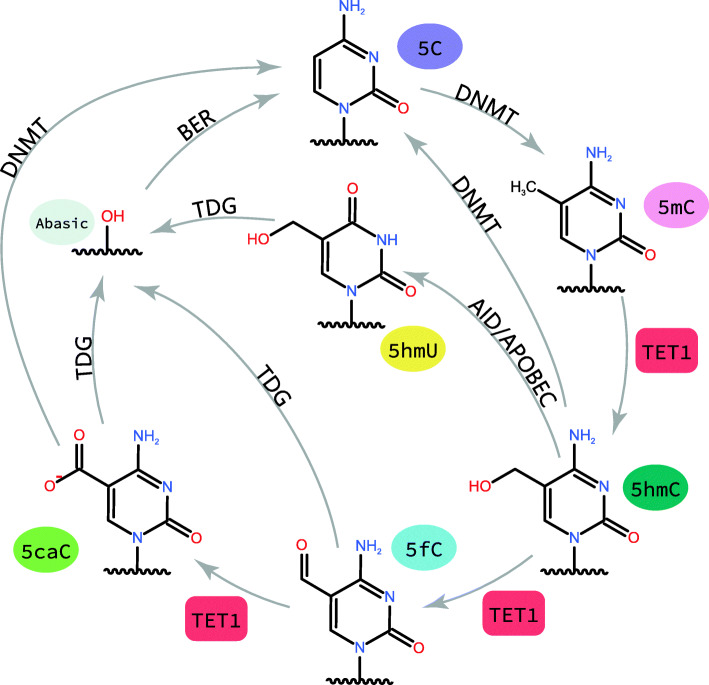
TET1-mediated active DNA demethylation. A complete model of TET1-mediated active DNA demethylation is shown. 5mC is formed by DNMT. TET1 gradually catalyze the oxidation of 5mC to form 5hmC, 5fC and 5caC. Among which 5hmC and 5caC can be eliminate the hydroxymethyl group and the carboxyl group to generate unmodified cytosine directly by DNMT, and 5hmC can also be catalyzed deamination by AID/APOBEC to 5hmU.5hmU along with 5fC and 5caC formed in the previous steps can be recognized and excised by TDG to generate an abasic site as part of the base excision repair (BER) process that regenerates unmodified C

To summarize, deaminases play a limited function in DNA demethylation relative to the oxidation-mediated pathway (Fig. 2).

### TET-mediated decarboxylation of 5caC

Direct decarboxylation of 5caC to achieve demethylation has a high energy barrier, but Schiesser S et al. [26] studied 5caC isotope labelling and demonstrated the feasibility of direct removal of oxidized amino acids; that is, 5caC can be directly decarboxylated and reduced to unmodified C in the presence of lysis products from embryonic stem cells. Oligonucleotides containing 5caC are synthesized and repaired in the presence of lysis products from embryonic stem cells, where the pyrimidine ring of 5caC is labelled by ^15^N. A small amount of ^15^N_2_-deoxycytidine was detected in the repair and disassembly of the oligonucleotide, indicating that 5caC can be directly decarboxylated to C in the absence of BER. Moreover, this pathway has a precedent for heterologous acid decarboxylase.

Zita Liutkeviciute et al. [27] demonstrated that both bacterial and mammalian DNMTs may catalyse the direct decarboxylation of 5caC to generate unmodified cytosine in DNA in vitro and can eliminate the hydroxymethyl group of 5hmC, directly converting 5hmC to unmodified C, but are inert to 5fC. Under simulated enzyme-catalysed conditions (high concentrations of exogenous thiols and imidazole, pH = 5.0), the C-C bond breakage of all three oxidized cytosines occurs in the following rate order: 5CaC > 5fC > 5hmC. Reducing conditions favour the onset of DNMT3a methyl transfer, whereas oxidizing conditions favour DNMT-mediated dehydroxymethylation. The atypical C-C bond cleavage reaction provides the possibility of direct “reversal” of 5caC to unmodified C. However, the direct decarboxylation of 5caC by 5caC decarboxylase in vivo has not been determined, and further studies are needed (Fig. 2).

## TET1 with stem cells and immunity

### TET1 with stem cells

TET1 and 5hmC have essential roles in maintaining the pluripotent state of embryonic stem cells (ESCs). In 2010 Shinsuke Ito et al. [3] studied mouse embryonic stem cells and found that TET1 has an important role in mouse embryonic stem (ES) cell maintenance by maintaining the expression of Nanog in ES cells. Downregulation of Nanog via TET1 knockdown correlates with methylation of the Nanog promoter, supporting a role for TET1 in regulating DNA methylation status. Furthermore, knockdown of TET1 in preimplantation embryos results in a bias towards trophectoderm differentiation. Their studies not only uncovered the enzymatic activity of TET proteins but also demonstrated a role for TET1 in ES cell maintenance and inner cell mass cell specification.

Subsequently, numerous studies have shown that TET1 binds primarily to the transcription start site of the CpG-rich promoter of the embryonic stem cell genome and regulates the level of DNA methylation in the promoter of the CpG-rich region. The competitive balance between the TET1 protein and DNMT at transcription start sites and other genomic loci promotes rapid changes with methylation status, activating or silencing transcription in a cell line and site-specific manner [28]. TET1 uses a two-pronged approach to maintain the self-renewal capacity and pluripotency potential of embryonic stem cells by “silencing” differentiation genes on the one hand and “activating” pluripotency genes on the other. The “silencing” of differentiation genes by TET1 is closely related to the transcriptional regulation of polycomb repressive complex 2 (PRC2). PRC2 recruits DNMT3a and DNMT3b to the promoters of genes involved in germ cell differentiation and mediates the enrichment of tri-methylation at lysine 27 of histone H3 (H3K27me3) to initiate de novo methylation [29, 30]. In the CpG-enriched region, DNA methylation prevents the binding of embryonic ectoderm development/enhancer of zeste 2/suppressor of zeste 12 (EED/EZH2/SUZ12, the core of the PRC2 subunit) to specific promoter regions of chromatin, whereas TET1 can preferentially bind to the CpG region, maintaining a hypermethylated state of the human embryonic stem cell promoter and ensuring that strong spectrum-specific transcription is maintained after differentiation by preventing ab initio methylation of the human embryonic stem cell promoter [31–33].

Notably, promoter DNA methylation does not immediately lead to transcriptional changes in human embryonic stem cells but can still impair their differentiation [3]. TET1 regulates many genes that define ectodermal and extraembryonic ectodermal differentiation programs. In ectodermal cells, TET1 demethylates gene promoters and maintains telomere stability through hydroxymethylation [34]. A series of studies have shown that knockdown of TET1 alone or co-knockdown of TET1 and TET2 in mouse embryonic stem cells leads to difficulties in maintaining embryonic stem cell pluripotency and skewed differentiation towards ectoderm and primitive endoderm [34]. TET1 maintains the “activation” of pluripotent genes by demethylation of gene promoters and represses most epistatic target genes independent of methylation changes by regulating the expression of the gene encoding the transcriptional repressor Jumonji domain-containing protein 8 (JMJD8) [34]. In primordial germ cell meiosis, DNA methylation, polycomb repressive complex 1 (PRC1) and TET1-mediated DNA demethylation activate a key set of germ cell reprogramming response genes involved in gamete production and meiosis [35].

Surprisingly, independent of methylation changes TET1 can also repress most ectodermal target genes, in part by regulating the gene encoding the transcriptional repressor JMJD8. In the absence of TET1, dysregulation of gene expression results in embryonic defects that are partially penetrant in inbred strains but completely lethal in noninbred mice [34].

In bone marrow mesenchymal stem cells (BMMSCs), TET1 plays a key role in maintaining BMMSC and bone homeostasis by controlling exosome and miRNA release through P2X purinoceptor 7 (P2RX7) demethylation, and could be a target for the development of new therapies for osteopenia [36].

Overall, the present study highlights the interplay between the catalytic and noncatalytic activities of TET1, which is essential for normal development.

### TET1 with immunity

There is growing evidence of a link between DNA methylation and antitumour immunity. Hao-Xiang Wu et al. [37] collected and integrated clinical cohorts from published studies with annotated response and survival data as well as matched mutation data. The predictive function of specific mutated genes was first tested in a discovery cohort, later validated in a validation cohort, and further investigated in The Cancer Genome Atlas (TCGA) dataset to examine the relationship between specific mutated genes and tumour immunogenicity and antitumour immunity. They found that among 21 key genes involved in regulating DNA methylation, the TET1 mutation (TET1-MUT) was enriched in patients who responded to immune checkpoint inhibitor (ICI) therapy in the discovery cohort (*p* = 0.003). In the discovery cohort (*n* = 519), patients with TET1-MUT and TET1-wild-type (TET1-WT) were enriched in objective response rate (ORR, 60.9% versus 22.8%, *P* < 0.001), durable clinical benefit (DCB, 71.4% versus 31.6%, *P* < 0.001) and progression-free survival (PFS, hazard ratio = 0.46 [95% confidence interval, 0.25 to 0.82], *P* = 0.008), which were significantly different. In the validation cohort (*n* = 1395), there was a significant overall survival (OS) advantage for TET1-MUT patients compared to TET1-WT patients (hazard ratio = 0.47 [95% confidence interval, 0.25 to 0.88], *P* = 0.019), which, importantly, was not associated with tumour mutation burden or high microsatellite instability; nor was it attributable to the TET1-MUT prognostic impact (*P* > 0.05 for both non-ICI treatment cohorts). In the TCGA dataset, TET1-MUT was strongly associated with higher tumour mutational burden and neoantigen load, as well as inflammatory patterns, immune features and immune-related gene expression in tumour-infiltrating T lymphocytes. In patients treated with ICI, TET1-MUT was strongly associated with higher ORR, better DCB, longer PFS and improved OS, suggesting that TET1-MUT is a novel predictive biomarker for immune checkpoint blockade against multiple cancer types.

Regulatory T (Treg) cells are essential for maintaining immune homeostasis, and TET1 and TET2 catalyse the conversion of 5mC to 5hmC in forkhead box P3 (FOXP3) to establish Treg cell-specific hypomethylation patterns and stable FOXP3 expression. Thus, TET1 and TET2 deficiency leads to FOXP3 hypermethylation, impaired Treg cell differentiation and function, and autoimmune diseases [38].

## TET1 in clinical disease

TET1 plays different roles in different diseases and subtypes, some of which are still controversial (Table 1).

**Table 1:** The expression level of TET1 in different malignant diseases and subtypes

Malignant Diseases	Subtypes	Expression Level of TET1
Leukemia	AML	Low
	T-ALL	High
Breast Cancer	HER2	Unchanged
	HRBC	Low
	Triple Negative	High
Lung Cancer	Adenocarcinoma	High
	Squamous Cell Carcinoma	High
Hepatocellular Carcinoma	–	Low
Cholangiocarcinoma	Intrahepatic Cholangiocarcinoma	Low
Colon Cancer	–	Low
Pancreatic Cancer	–	Low
Prostate Cancer	–	Low
Cervical Cancer	–	High

### TET1 in leukaemia

TET1 was initially identified as a fusion protein in a specific case of acute myeloid leukaemia (AML) and was therefore presumed to be associated with leukaemogenesis. Subsequently, some AML patients were found to have an MLL-TET1 fusion gene, including the carboxyl terminus of TET1 (containing the 2-OG-Fe2+ oxidase structural domain) and the amino terminus of mixed lineage leukaemia (MLL) (containing the CXXC structural domain) [39], playing an important oncogenic role by activating homeobox gene expression.

In 2020 Tingjuan Zhang et al. [40] identified that TET1 expression was significantly reduced in AML patients but was highly expressed in T-cell acute lymphoblastic leukaemia (T-ALL) and essential for its development. In 2021, Shiva Bamezai et al. [41] reported that TET1 is highly expressed in T-ALL and is crucial for human T-ALL cell growth in vivo. Knockout of TET1 in mice and knockdown in human T cells did not perturb normal T-cell proliferation, indicating that TET1 expression is dispensable for normal T-cell growth. The promotion of leukaemic growth by TET1 was dependent on its catalytic property to maintain global 5-hydroxymethylcytosine (5hmC) levels, thereby regulating the cell cycle, DNA repair genes, and T-ALL associated oncogenes. Furthermore, overexpression of the TET1 catalytic domain was sufficient to augment global 5hmC levels and leukemic growth of T-ALL cells in vivo. Their study demonstrated that PARP enzymes, which are highly expressed in T-ALL patients, participate in establishing H3K4me3 marks at the TET1 promoter and that PARP1 interacts with the TET1 protein. Importantly, the growth-related role of TET1 in T-ALL could be antagonized by the clinically approved PARP inhibitor olaparib, which abrogated TET1 expression, induced loss of 5hmC, and antagonized leukaemic growth of T-ALL cells, opening a therapeutic avenue for this disease.

The TET protein family often displays opposite expression trends in different types of haematological diseases [40, 42]. TET2 mutations are frequent in patients with AML, myelodysplastic syndromes (MDS) and chronic myelocytic leukaemia (CML), while TET1 and TET3 mutations are rare [42, 43]. The mechanisms of which have yet to be studied in depth.

### TET1 in solid tumours

The global decrease in 5hmC with downregulation of TET and/or functional alterations in TET was once considered a hallmark of cancer, and this dysregulation of DNA methylation levels was found in various solid tumours, such as breast, lung, liver, colon, pancreatic and prostate cancers [44–47]. However, the latest studies have raised the possibility that TET1 can be both an oncogene and tumour suppressor gene.

#### TET1 in breast cancer

High or low DNA methylation levels are found in breast cancer, but factors regulating the balance of methylation and demethylation remain unclear. However, that the degree of DNA demethylation in breast cancer is closely related to the growth, invasion, and metastasis of breast cancer cells.

In 2012, Chih-Hung Hsu et al. [44] found that TET1 was suppressed in breast cancer tissues. The depletion of TET1 in xenograft models promoted breast cancer cell invasion and growth and induced breast cancer cell metastasis. Overexpression of TET1 inhibits tumour cell invasion and xenograft tumour formation by activating tissue inhibitors of metalloproteinases. In 2018, Naifei Chen et al. [48] found that an exonic circular RNA (FLI1 exonic circular RNA, FECR1) regulates the balance of methylation and demethylation regulating breast cancer growth and promoting breast cancer metastasis by recruiting TET1 together with transDNMT1.

Triple-negative breast cancer (TNBC), a subtype that does not overexpress estrogen receptor, progesterone receptor or human epidermal growth factor receptor 2 (HER2) gene (ER^−^/PR^−^/HER2^−^), is a minimally methylated cancer. In 2018, Charly Ryan Good et al. [49] compared of 100 TNBC patients and 732 hormone receptor positive (HRBC) patients with 105 normal subjects. They showed that TET1 expression was significantly increased in TNBC compared to normal breast. Through bioinformatic analyses in both breast and ovarian cancer cell line panels, they uncovered an intricate network connecting TET1 to hypomethylation and activation of cancer-specific oncogenic pathways, including phosphatidylinositol 3-kinase (PI3K), epidermal growth factor receptor (EGFR), and platelet derived growth factor (PDGF). TET1 expression correlated with sensitivity to drugs targeting the PI3K-mTOR pathway, and CRISPR-mediated deletion of TET1 in two independent TNBC cell lines resulted in reduced expression of PI3K pathway genes, upregulation of immune response genes, and substantially reduced cellular proliferation, suggesting dependence of oncogenic pathways on TET1 overexpression. Their work establishes TET1 as a potential oncogene that contributes to aberrant hypomethylation in cancer and suggests that TET1 could serve as a druggable target for therapeutic intervention.

In 2019 Yong Yu et al. [50] confirmed that in TNBC cells, EZH2 inhibited TET1 expression and subsequently inhibited the antitumour p53 signalling pathway, and patients with high EZH2 expression and low TET1 expression had the shortest survival. Targeted suppression of EZH2 in TNBC cells in vitro using the specific inhibitor GSK343 or shRNA can induce cell cycle arrest and cellular senescence by increasing TET1 expression and p53 pathway activation. This study opens a new avenue for TNBC treatment by targeting the EZH2-H3K27me3-TET1 pathway, which can modulate the epigenetic landscape. In 2020, Bin Bao et al. [51] demonstrated that TET1- and TET1-dependent 5hmC mediated a novel hydrogen peroxide-(H_2_O_2_)-dependent gene expression cascade response driving the self-renewal and expansion of cancer stem cell-like cells (CSCs) in TNBC.

According to the study of Charly Ryan Good, the expression of TET1 was significantly suppressed in HRBC patients while unchanged in HER2-positive cases. This differential result suggests that the degree of TET1 expression varies among different subtypes of breast cancer. Hence, it is necessary to fully consider different subtypes of breast cancer when exploring therapeutic targets for breast cancer based on the suppressive effect of TET1 on related genes.

#### TET1 in lung cancer

The role TET1 plays in lung cancer remains controversial. In 2016 Matteo Forloni et al. [52] demonstrated that EGFR triggered the silencing of multiple tumour suppressors in lung adenocarcinoma via C/EBPα transcription factor by inhibiting TET1 expression. After EGFR inhibition, TET1 binds to the promotor of the tumour suppressor genes and induces its repression through DNA demethylation. Ectopic expression of TET1 effectively inhibits the growth of the lung cancer, while knockdown of TET1 causes lung adenocarcinoma cells to develop resistance to EGFR inhibitors. Usually, TET1 in lung cancer showed decreased expression or cytoplasmic localization. In 2017, a study conducted by J-I Lai et al. [53] found that patients with higher TET1 levels showed a trend towards increased response efficiency to EGFR inhibitors compared to patients with lower TET1 expression, but the trend was not significant (*p* = 0.08). Furthermore, no correlation was observed between TET1 expression levels and patient survival. Although the oncogenic role of TET1 inhibition against EGFR has been established in cellular and animal models of lung cancer, its role in patient prognosis is still inconclusive and worth further investigation.

In 2019 Piotr T Filipczak et al. [54] found overexpression of TET1 in lung adenocarcinoma and squamous cell carcinoma. They demonstrated that knockdown of TET1 inhibited cell growth in vitro and in vivo and induced transcriptome reprogramming, affecting critical signalling pathways in cancer, but this effect was independent of its demethylation activity. Wild-type p53 binds to the promoter of TET1 to repress transcription, and p53 mutations are closely associated with high TET1 expression. Knockdown of TET1 in p53 mutant cell lines successfully induced cellular senescence. These data identify TET1 as a proto-oncogene in lung cancer, and its enhanced function following p53 deletion may be exploited by targeted therapy-induced senescence. In 2021 Hong-Qiang Chen et al. [55] found that TET1 expression was significantly downregulated in 3-methylcholanthrene (3-MCA)-induced cellular malignant transformation model, rat chemical carcinogenesis model and human lung cancer tissues. Both in vitro and in vivo experiments showed that TET1 overexpression inhibited lung cancer cell proliferation, migration and invasion, while knockdown of TET1 resulted in the opposite effect. In malignantly transformed cells, the promoters of XRCC1, human 8-ox-oguanine DNA glycosylase 1 (OGG1), and DNA-(apurinic or apyrimidinic site) endonuclease (APEX1), key genes of the BER pathway, showed significantly lower levels of DNA hydroxymethylation and significantly higher levels of methylation. After differential expression of TET1, the hydroxymethylation, methylation and expression levels of these genes were also significantly altered. In addition, TET1 binds to XRCC1, OGG1 and APEX1 to maintain their hydroxymethylation, and blocking one or several essential genes in the BER pathway significantly inhibits the effect of TET1. This study demonstrates for the first time that TET1 expression is regulated by DNA methylation during 3-MCA-induced lung carcinogenesis and that TET1-mediated hydroxymethylation regulates the BER pathway and inhibits cancer cell proliferation, migration and invasion.

#### TET1 in hepatocellular carcinoma and cholangiocarcinoma

The role of TET1 in hepatocellular carcinoma has not been thoroughly investigated. Available studies have confirmed that TET1 expression is suppressed in hepatocellular carcinoma (HCC), cells cultured in vitro, and some rapidly proliferating hepatocytes after hepatectomy [56], yet there is no significant change in TET1 transcript levels [56, 57].

The progression of cholangiocarcinoma (CCA) was highly correlated with TET1. Knockdown of TET1 can epigenetically inhibit CCA progression through targeted regulation of cell growth and apoptosis. In a liver in situ xenograft model, suppressing TET1 expression remarkably inhibited CCA malignant progression by inhibiting cell growth and inducing apoptosis [58]. TET1 decreases in intrahepatic cholangiocarcinoma (ICC) and suppresses ICC progression by activating oncogenes through direct binding to oncogene promoters for demethylation [59].

#### TET1 in colon cancer

DNA methylation abnormalities are common in colon cancer (CC). Current studies suggest that TET1 acts as a suppressor of CC through the Wnt signalling pathway. In 2015, F Neri et al. [60] considered TET1 as a tumour suppressor that inhibits colon cancer growth by antagonizing inhibitors of the Wnt signalling pathway. TET1 binds to the promoter of the inhibitor of the DKK gene, one of the components of the Wnt signalling pathway, to maintain its hypomethylation. In 2019, Hailong Guo et al. [61] demonstrated that TET1 inhibited CC proliferation by suppressing the β-catenin signalling pathway.

#### TET1 in pancreatic cancer

Similar to CC, TET1 inhibits pancreatic cancer progression by blocking the Wnt signalling pathway and suppresses pancreatic tumour proliferation and metastasis in vivo and in vitro. TET1 binds to the secreted frizzled-related protein 2 (SFRP2) promoter and catalyses its demethylation to activate SFRP2 transcription, thereby repressing the Wnt/β-catenin signalling pathway and ultimately restraining the epithelial-mesenchymal transition (EMT) in pancreatic tumours. Pancreatic cancer patients with low TET1 expression levels have shorter survival rates than those with higher TET1 levels [62].

TET1 suppresses the Hedgehog signalling pathway through demethylation of CHL1 to downregulate EMT and chemoresistance in pancreatic ductal adenocarcinoma [63].

#### TET1 in prostate cancer

TET1 is downregulated in prostate cancer tissues. In xenograft models, TET1 depletion promotes tumour cell growth, invasion and metastasis [64]. TET1 inhibits prostate cancer invasion by activating tissue inhibitors of metalloproteinases [44].

#### TET1 in cervical cancer

In 2019, Po-Hsuan Su et al. [65] found increased TET1 and 5hmC correlation from normal to low-grade squamous intraepithelial lesions (LSIL) in the cervix, maximized in high-grade squamous intraepithelial lesions (HSIL), and reduced in invasive carcinoma. Full-length HPV-immortalized HSIL cells demonstrated higher TET1/5hmC levels and stemness properties than invasive cancer cells. TET1 silencing promoted EMT to transform precancerous cells in vivo. TET1 increased 5hmC in the zinc finger E-box binding homeobox 1 (ZEB1) and Vimentin (VIM) promoters; surprisingly, both genes were silenced. TET1 interaction with the histone modifiers lysine specific demethylase 1 (LSD1) and EZH2 on the ZEB1 promoter resulted in gene silencing via loss of tri-methylation at histone lysine 4 of histone (H3K4me3) and gain of histone H3K27me3. Taken together, TET1 promotes stemness properties and inhibits EMT in HSIL cells through 5hmC-dependent and 5hmC-independent mechanisms. Recently, several studies have proposed that TET1 interacts with the immune system to influence the epigenetics of cancer cells. In 2019, Hao-Xiang Wu et al. [37] collected and consolidated clinical cohorts with annotated response and survival data and matched mutational data from published studies. The predictive function of specific mutated genes was first tested in the discovery cohort and later validated in the validation cohort. The association between specific mutated genes and tumour immunogenicity and antitumour immunity was further investigated in TCGA dataset. TET1 was recurrently mutated across multiple cancers and more frequently seen in skin, lung, gastrointestinal, and urogenital cancers. Collignon et al. [45] demonstrated in their study of basal-like breast cancer (BLBC) that TET1 inhibition was associated with high expression of immune markers and high infiltration of immune cells. In BLBC tissues, there was a negative correlation between TET1 expression and nuclear factor κB (NF-κB), the main immune regulator family.

### TET1 in metabolism

TET1 is an autonomous blocker of critical thermogenic genes (including UCP1 and PPARGC1a) in brown adipocytes. TET1 knockout mice showed improved cold tolerance, increased energy expenditure, and suppression of diet-induced obesity and insulin resistance. Furthermore, the repressive role of TET1 in thermogenic gene regulation in brown adipocytes is largely independent of DNA demethylase but coordinates with the proto-oncogene histone deacetylase 1 (HDAC1) to mediate epigenetic changes to repress thermogenic gene transcription [66].

Nonalcoholic fatty liver disease (NAFLD) is a lipid metabolism disease and an important driver of cirrhosis and hepatocellular carcinoma. TET1 promotes fatty acid oxidation and inhibits NAFLD progression through hydroxymethylation of the peroxisome proliferator-activated receptors α (PPARα) promoter [67, 68].

TET1 stabilizes hypoxia-inducible factor α (HIF-α) and enhances HIF-α transcription activity independent of its enzymatic activity. TET1 competes with prolyl hydroxylase protein 2 (PHD2) to bind to HIF-2α, resulting in a reduction in HIF-2α hydroxylation by PHD2. However, TET1 has no effect on HIF-1α hydroxylation, but rather it appears to stabilize the C-terminus of HIF-1α by affecting lysine site modification.

### TET1 in the neurological system

TET1 is closely associated with psychiatric disorders (e.g., schizophrenia, autism). Upregulation of TET1 expression in the genic parietal cortex of patients with psychiatric disorders increases the level of 5hmC in promoter region 67 of brain glutamic acid decarboxylase, induces activation of AID/APOBEC expression, and downregulates and impairs the 5hmU base excision repair pathway. These findings may play a role in the pathophysiological mechanisms of certain psychiatric disorders [69]. TET1 isoforms differentially regulate gene expression, synaptic transmission and memory in the mammalian brain [70, 71].

Life experiences can leave lasting marks in the brain, such as epigenetic changes. How life experiences are translated into storable epigenetic information remains largely unknown. Current studies have demonstrated that early growth response 1 (EGR1) recruits TET1 to remove methylation marks and activate downstream genes, providing new insight into how life experiences may shape the brain methylome [72].

TET1 has recently been suggested to be linked to pain hypersensitivity, TET1 demethylates the mGluR5 promoter to cause pain, and this effect can be alleviated by melatonin [73].

## Conclusions

DNA demethylation is an important aspect of epigenetic modification and TET1 plays an important role in this process. Although the exact mechanism has yielded promising results with advanced studies, some details have still not been clarified. For example, the direct decarboxylation of 5caC decarboxylase in vivo has not been determined, and the interaction between TET1 and DNMT in the process of DNMT-mediated decarboxylation still deserves further exploration. In addition, the role of TET1 in tumours remains controversial. The loss of function of CpG islands in the promoter due to abnormal hypermethylation is an important pathway for antioncogene inactivation. In most tumours, TET1 reverses the inactivation of hypermethylated antioncogenes and acts as an “activator”. Recent studies suggest that TET1 is differentially expressed in different types of tumours and different subtypes of the same tumour and exhibits different biological effects by activating different genes in different signalling pathways, thus displaying both oncogene and antioncogene properties. However, the exact mechanism needs to be further clarified. The research of regarding TET1 as a potential therapeutic target has also made some progress, especially in T-ALL, TNBC and lung adenocarcinoma. But its complex role in different signal pathways has limited its application. TET1 has been studied in stem cells, neuroregeneration, metabolism, ageing, and psychiatric diseases but not yet in depth. In recent years, TET1 has also attracted attention due to its noncatalytic role in early neuronal differentiation. In conclusion, research on the TET1 protein will provide a wider range of ideas for epigenetic studies, mechanisms of related diseases and new therapeutic targets. Research on the TET1 protein has broad prospects and is of great value in medicine.

## Data Availability

Not applicable.

## Abbreviations

TET: Ten-eleven translocation
5mC: 5-methylcytosine
DNMT: DNA methyltransferase
SAM: S-adenosyl methionine
5C: 5-cytosine
CD: Cysteine-rich domain
CXXC: Cys-Xaa-Xaa-Cys
DSBH: Double-stranded b-helix
5hmC: 5-hydroxymethylcytosine
UHRF1: Ubiquitin-like with PHD and ring finger domains 1
BER: Base excision repair
TDG: Thymidine-DNA glycosylase
5fC: 5-Formylcytosine
5caC: 5-carboxylcytosine
XRCC1: X-ray repair cross complementary protein 1
LIG3: DNA ligase 3
POLb: DNA polymerase b
AID: Activation-induced deaminase
APOBEC: Apolipo protein B mRNA-editing enzyme-catalyzed polypeptide-like
5hmU: 5-hydroxymethyluracil
AP: Apurinic/apyrimidinic
APE1/Ref-1: Apurinic aprimidinic endonuclease/Redox factor-1
GADD45: Growth arrest and DNA damage inducible 45
MBD4: Growth arrest and DNA damage inducible 45
dsDNA: Double-stranded DNA
ESCs: Embryonic stem cells
ES: Embryonic stem
PRC2: Polycomb repressive complex 2
H3K27me3: Tri-methylation at lysine 27 of histone H3
EED: Embryonic ectoderm development
EZH2: Enhancer of zeste 2
SUZ12: Suppressor of zeste 12
JMJD8: Jumonji domain-containing protein 8
PRC1: Polycomb repressive complex 1
BMMSCs: Bone marrow mesenchymal stem cells
P2RX7: P2X purinoceptor 7
TCGA: The Cancer Genome Atlas
ORR: Objective response rate
DCB: Durable clinical benefit
PFS: Progression-free survival
OS: Overall survival
Treg: Regulator T
FOXP3: Forkhead box P3
AML: Acute myeloid leukemia
MLL: Mixed lineage leukemia
T-ALL: T-cell acute lymphoblastic leukemia
MDS: Myelodysplastic syndromes
CML: Chronic myelocytic leukemia
FECR1: FLI1 exonic circular RNA
TNBC: Triple-negative breast cancer
HER2: Human epidermal growth factor receptor 2
HRBC: Hormone receptor positive
PI3K: Phosphatidylinositol 3-kinase
EGFR: Epidermal growth factor receptor
PDGF: Platelet derived growth factor
CSCs: Cancer stem cell-like cells
3-MCA: 3-methylcholanthrene
OGG1: Human 8-ox-oguanine DNA glycosylase 1
APEX1: DNA- (apurinic or apyrimidinic site) endonuclease
HCC: Hepatocellular carcinoma
CCA: Cholangiocarcinoma
ICC: Intrahepatic cholangiocarcinoma
CC: Colon cancer
SFRP2: Secreted frizzled-related protein 2
EMT: Epithelial-mesenchymal transition
LSIL: Low-grade squamous intraepithelial lesions
HSIL: High-grade squamous intraepithelial lesions
ZEB1: Zinc finger E-box binding homeobox 1
VIM: Vimentin
LSD1: Lysine specific demethylase 1
H3K4me3: Tri-methylation at lysine 4 of histone H3
BLBC: Basal-like breast cancer
NF-κB: Nuclear factor κB
HDAC1: Histone deacetylase 1
NAFLD: Non-alcoholic fatty liver disease
PPARα: Peroxisome proliferator-activated receptors α
HIF-α: Hypoxia-inducible factor α
PHD2: Prolyl hydroxylase protein 2
EGR1: Early growth response 1
